# Identification and Validation of Potential Biomarkers for the Detection of Dysregulated microRNA by qPCR in Patients with Colorectal Adenocarcinoma

**DOI:** 10.1371/journal.pone.0120024

**Published:** 2015-03-24

**Authors:** Xiaobing Wu, Xuehu Xu, Shuling Li, Shangbiao Wu, Rong Chen, Qingping Jiang, Haibo Liu, Yan Sun, Yong Li, Yuandong Xu

**Affiliations:** 1 Department of Gastrointestinal Surgery, Third Affiliated Hospital of Guangzhou Medical University, Guangzhou, China; 2 Department of Pathology, Third Affiliated Hospital of Guangzhou Medical University, Guangzhou, China; 3 Department of Experimental Research, Third Affiliated Hospital of Guangzhou Medical University, Guangzhou, China; 4 Department of Gastroenterology, Third Affiliated Hospital of Guangzhou Medical University, Guangzhou, China; Queen Mary Hospital, HONG KONG

## Abstract

Colorectal cancer represents a lethal disease that has raised concern and has attracted significant attention. Adenocarcinoma is the most common type of colorectal cancer (CRC). MicroRNAs are thought to be potential biomarkers of CRC. Many researchers have focused on the expression pattern of miRNAs in CRC. However, previous studies did not pay particular attention to the effects of the degree of differentiation of the cancer with respect to the miRNA expression profile. First, this study compared the expression level of 1547 miRNAs by qRT-PCR in Colorectal adenocarcinoma tissues to that in paired normal tissues. In all, 93 miRNAs were identified that were significantly dysregulated in Colorectal adenocarcinoma relative to normal tissues (*P*<0.05). Then, we analyzed their potential as cancer biomarkers by ROC analysis, and the result revealed that three miRNAs with high sensitivity and specificity are suitable as biomarkers for the diagnosis of CRC (the value of the AUC was greater than 0.7). Interestingly, previous reports of 23 of these miRNAs have been scarce. Furthermore, we wanted to analyze the difference between well- and moderately differentiated cancers, and as expected, 58 miRNAs showed significant dysregulation. Importantly, 32 miRNAs were able to not only distinguish cancer tissues from normal tissues, but they were also able to identify well- and moderately differentiated cancers. In conclusion, the degree of differentiation has an important influence on the miRNA expression pattern. To avoid misdiagnoses and missed diagnoses, tumors of different degrees of differentiation should be treated differently when miRNAs are used as cancer biomarkers.

## Introduction

Colorectal cancer is a heavy health burden and is estimated to be the third most common cancer worldwide in both sexes [1–3]. In fact, it has been estimated that CRC caused more than 500,000 deaths worldwide in 2013 alone [4]. Current screening tools for CRC are still unsatisfactory because of low sensitivity and specificity. Therefore, many researchers have attempted to identify novel biomarkers for the detection of CRC, and many studies have found that microRNAs (miRNAs) are dysregulated in many cancer types include CRC [5,6]. Therefore, miRNAs have the potential to serve as diagnostic biomarkers for CRC [7].

MiRNAs are a class of short non-coding RNA molecules that regulate gene expression at the post-transcriptional level [8]. Since the identification of miRNAs in 1993 [9], the number of identified miRNAs has already reached 2578 according to miRBase (V20). Many studies have previously shown that miRNAs may be involved in the process of tumorigenesis and may act as either tumor suppressors or oncogenes [10]. Hence, miRNAs may be used as biomarkers for the clinical diagnosis of colorectal tumors. However, while most previous studies focused on the different expression levels of miRNAs between normal controls and patients with cancer [11], we have focused on the differences among the various stages of CRC [12]. However, to our knowledge, no study has reported the difference in miRNA expression according to the degree of differentiation of CRC. As is known, CRC includes a variety of differentiated types. We believe it is important to know the effects of the degree of differentiation in the study of the expression of miRNAs that are used as biomarkers.

This study first made an effort to analyze the miRNA profiles according to the degree of differentiation of the cancer; we expected to identify and validate some suitable miRNAs for the detection of CRC. In the present study, we organized a profile of 1547 distinct human miRNAs found in paired tumor and adjacent normal mucosa samples obtained from 28 patients with colorectal adenocarcinoma; we then compared the miRNA profiles between well- and moderately differentiated tumors.

## Materials and Methods

### Patients and specimens

The Clinical Research Ethics Committee of the Third Affiliated Hospital of Guangzhou Medical University approved the research protocols, and written informed consent was provided by the participants. Demographic information was obtained from the patient records and registries (Table 1). Fifty-six tissue specimens, including 28 tumor tissues and 28 paired adjacent normal mucosa samples, were selected from 28 cases of Colorectal adenocarcinoma. These samples consisted of 21 cases of moderately differentiated adenocarcinoma and 7 cases of well-differentiated adenocarcinoma. In addition, 12 CRC specimens and paired normal samples were collected for an independent sample test to confirm the previous microRNA profiling results. All of these patients underwent surgery at the Third Affiliated Hospital of Guangzhou Medical University. Patients who received preoperative chemotherapy or radiation therapy or who had a previous history of malignant tumors were excluded. All patients with colorectal adenocarcinoma were diagnosed at the Department of Pathology, Third Affiliated Hospital of Guangzhou Medical University, with a final pathological diagnosis of CRC. The tissue samples were flash-frozen in liquid nitrogen after resection and stored at -80°C until the nucleic acids were extracted.

**Table 1:** Clinical and histopathological characteristics of patients.

Patient ID	Age (y)	Gender	Differentiation	Anatomic site	Cancer type
1	72	Male	Moderate	Colon	Adenocarcinoma
2	71	Male	Moderate	Rectum	Adenocarcinoma
3	45	Male	Moderate	Colon	Adenocarcinoma
4	56	Male	Moderate	Rectum	Adenocarcinoma
5	74	Female	Well	Colon	Adenocarcinoma
6	83	Female	Moderate	Rectum	Adenocarcinoma
7	38	Male	Moderate	Rectum	Adenocarcinoma
8	20	Female	Moderate	Colon	Adenocarcinoma
9	73	Male	Moderate	Colon	Adenocarcinoma
10	77	Female	Moderate	Colon	Adenocarcinoma
11	61	Male	Moderate	Rectum	Adenocarcinoma
12	60	Female	Moderate	Colon	Adenocarcinoma
13	60	Male	Moderate	Rectum	Adenocarcinoma
14	72	Female	Well	Rectum	Adenocarcinoma
15	38	Male	Well	Colon	Adenocarcinoma
16	71	Female	Moderate	Colon	Adenocarcinoma
17	74	Female	Moderate	Rectum	Adenocarcinoma
18	53	Female	Moderate	Rectum	Adenocarcinoma
19	77	Male	Moderate	Colon	Adenocarcinoma
20	77	Female	Well	Colon	Adenocarcinoma
21	73	Female	Well	Colon	Adenocarcinoma
22	57	Female	Moderate	Colon	Adenocarcinoma
23	70	Female	Well	Colon	Adenocarcinoma
24	50	Male	Moderate	Colon	Adenocarcinoma
25	54	Female	Moderate	Colon	Adenocarcinoma
26	77	Male	Moderate	Rectum	Adenocarcinoma
27	74	Female	Moderate	Rectum	Adenocarcinoma
28	82	Male	Well	Colon	Adenocarcinoma

### RNA isolation

Frozen tissues (about 100 mg) were used to isolate miRNA with RNAzol reagent (Molecular Research Center,USA), according to the manufacturer’s instructions. Briefly, 100 mg of tissue was homogenized with 1 mL of RNAzol. To form the homogenates, 0.4 mL water was added. After 5–10 min, the mixture was centrifuged at 12 000*g for 15 min in order to precipitate the DNA/protein. One milliliter of the supernatant was mixed with 0.4 mL of 75% ethanol for 10 min and centrifuged at 12 000*g for 8 min in order to precipitate the miRNA. The collected miRNA supernatant was mixed with isopropanol (0.8 volumes) for 30 min and then centrifuged at 12 000* g for 15 min, washed 26 with 0.4 mL 70% isopropanol, and centrifuged at 8000*g for 3 min. Finally, the miRNAs were dissolved in diethylpyrocarbonate-treated water. Electrophoresis and a NanoDrop spectrophotometer (Thermo Scientific, USA) were used to evaluate the concentration and purity of the miRNAs. The quality of the miRNAs was considered acceptable if the OD260/OD280 was between 1.7 and 2.0. The integrity of small RNAs was evaluated by the determination of robust amplification of small nuclear ubiquitous RNAs (i.e., RNU6b, RNU44, and RNU48) by real-time reverse-transcription PCR (qRT-PCR) because they are commonly used as endogenous controls in miRNA studies.

### miRNA profiling

A Universal RT microRNA PCR system (GeneCopoeia, USA) was applied to profile the miRNAs using two pooled tissue samples (i.e., 28tumors and 28 paired normal controls). The profiling assay included a universal reverse transcription (RT) and sequential qRT-PCR amplification with special primers using SYBR Green.

### Reaction

The PCR reaction was performed in a 384-well PCR plate, and each well contained 20 μL of the reaction system, including 1 μL of cDNA and 1 μL of gene-specific PCR primers. A total volume of 50 mL of the PCR reaction system including 2.5 mL of cDNA of each tissue sample for 9*384-well plates was selected and uniformly mixed. A total of 1547 distinct miRNAs was used for the profiling, and the fold changes in the expression of each type of miRNA were determined.

### Quantification of miRNAs by qRT-PCR

miRNAs (approximately 500 ng) were reverse transcribed in 25-mL reaction volumes with an All-in-One First-Strand cDNA Synthesis kit (GeneCopoeia, USA). In brief, 25 μL of RT reaction mix included the miRNA sample, 5 μL of 5x reaction buffer, 2.5 U/μL PolyA Polymerase, 10 ng/μL MS2 RNA, and RTase Mix. The reaction was performed at 37°C for 60 min and terminated at 85°C for 5 min. cDNA that was produced in the RT reaction was diluted ten-fold and was used as the template for the PCR reaction in an Applied Biosystems ViiA 7 Real-Time PCR System (Life Technologies, USA). In this system, MS2 RNA was used as an external reference for the quality of the extracted miRNAs, and RNU6B, RNU44, RNU48, and RNU49 were used for normalization. The PCR system contained the following components within a total volume of 20 μL per well: 1 μL template, 2 μL PCR forward primer (2 μM), 2 μL PCR reverse primer (2 μM), 10 μL 2x All-in-one qPCR Mix, 4.8 μL ddH2O and 0.2 mL 50x ROX Reference Dye (for calibration). A master mix was often prepared first, which included all of the components except for the template. If the total volume of the master mix changed, each component was added in the proper proportion. The conditions of real-time PCR were as follows: hot-start denaturation at 95°C for 10 min, 40 cycles of amplification with denaturation at 95°C for 10 s, annealing at 60°C for 20 s, and extension at 72°C for 15 s, then melting at 60°C for 10 min and finally cooling at 25°C for 30 s. To optimize the PCR conditions, a preliminary experiment was performed to ensure that the difference in the cycle threshold (CT) value of the MS2 RNA between the cancer samples and paired normal samples did not exceed 1.0. The expression levels of the miRNAs were quantified by SYBR Green-based All-in-One qPCR Mix (GeneCopoeia, USA).

### Statistical analyses

SPSS 16.0(SPSS, UK) and Graph Pad Prism 5.0 (San Diego, CA, USA) software were used for the data analyses. The comparative expression of miRNAs was determined by the 2^-ΔΔCT^ method [13] and non-paired-t test as well as by analysis of the receiver operating characteristic (ROC) curves. The area under the ROC curve (AUC) was used for the evaluation of the sensitivity and specificity of the miRNAs from the tissue specimens as a diagnostic marker for the detection of Colorectal adenocarcinoma. In the two-tailed test, a *P*-value<0.05 was considered statistically significant.

## Results

Compared with normal tissues, a total of 93 miRNAs that were significantly dysregulated were identified by non-paired t-test, of which 82 were downregulated and 11 were upregulated (Table 2). With the exception of miR-150, miR-181a, miR-130b, and let-7i, which demonstrated less than a 2-fold change relative to the normal controls, the fold changes of the other 89 dysregulated miRNAs were greater than 2-fold (Table 2). Furthermore, among the dysregulated miRNAs, the fold changes of miR-1, miR-145 and miR-145* were downregulated more than 10-fold. Others that were downregulated more than 5-fold included miR-137, miR-133a, miR-4770, miR-143, miR-363, miR-490–5p, miR-133b, let-7d, miR-4510, miR-9, miR-144*, miR-99b, miR-99a, miR-4469 and miR-451. Out of the miRNAs that were upregulated, the fold change of miR-135b, miR-96 and miR-141 was more than five-fold (Table 2). Interestingly, to our knowledge, 23 of these miRNAs have never been reported to be dysregulated in Colorectal adenocarcinoma (Table 3); among these miRNAs, the fold changes of miR-99b and miR-4469 were downregulated more than 5-fold.

**Table 2:** Dysregulated miRNAs in cancer tissues compared with normal controls by non-paired t-test.

miRNA	fold change	P_Value	miRNA	fold change	P_Value	miRNA	fold change	P_Value
miR-1	-17.241	0.000	let-7b	-3.623	0.003	miR-10b	-2.577	0.016
miR-145	-16.393	0.000	miR-328	-3.623	0.003	miR-140–3p	-2.558	0.016
miR-145*	-14.706	0.000	miR-365	-3.484	0.003	miR-23a	-2.545	0.017
miR-137	-9.901	0.000	miR-193a-5p	-3.460	0.003	miR-1979	-2.519	0.019
miR-133a	-8.403	0.000	miR-28–5p	-3.401	0.003	miR-30d*	-2.519	0.019
miR-4770	-7.407	0.000	miR-193b	-3.390	0.003	miR-30c	-2.392	0.020
miR-143	-7.194	0.000	miR-374a	-3.390	0.003	miR-10a	-2.342	0.021
miR-363	-6.667	0.000	miR-574–3p	-3.300	0.004	miR-29c	-2.315	0.021
miR-490–5p	-6.623	0.000	miR-9*	-3.268	0.004	miR-130a	-2.288	0.021
miR-133b	-6.452	0.000	miR-378i	-3.185	0.004	miR-30b	-2.257	0.022
let-7d	-5.952	0.000	miR-378*	-3.145	0.004	miR-409–5p	-2.222	0.023
miR-4510	-5.808	0.001	miR-30a	-3.145	0.005	miR-26a-2*	-2.222	0.025
miR-9	-5.780	0.000	miR-4423–3p	-3.125	0.005	miR-487b	-2.212	0.026
miR-99b	-5.566	0.001	miR-204	-3.086	0.005	miR-27b*	-2.198	0.026
miR-99a	-5.348	0.001	miR-27b	-3.058	0.006	miR-4443	-2.123	0.028
miR-4469	-5.222	0.002	miR-26b	-3.030	0.006	miR-181c	-2.058	0.028
miR-451	-5.051	0.001	miR-628–3p	-3.003	0.006	miR-4790–5p	-2.058	0.028
miR-143*	-4.854	0.001	miR-26a	-2.985	0.007	miR-150	-1.976	0.030
miR-144*	-4.747	0.000	miR-30c-2*	-2.959	0.007	miR-181a	-1.972	0.032
miR-23b	-4.630	0.001	miR-126	-2.950	0.009	let-7i	-1.876	0.032
miR-100	-4.608	0.001	miR-29b-2*	-2.950	0.009	miR-130b	1.870	0.036
miR-3195	-4.505	0.001	miR-195	-2.950	0.009	miR-200a	2.265	0.037
miR-125b	-4.386	0.001	let-7d*	-2.924	0.009	miR-203	2.417	0.037
miR-768–3p	-4.219	0.001	let-7g	-2.817	0.009	miR-429	2.772	0.037
miR-125a-5p	-4.184	0.001	miR-422a	-2.793	0.010	miR-592	2.946	0.038
miR-24–1*	-4.132	0.002	miR-374b	-2.710	0.010	miR-183	3.225	0.040
miR-30e*	-4.049	0.002	miR-214*	-2.695	0.010	miR-1290	3.341	0.044
miR-218	-4.000	0.002	miR-199b-3p	-2.646	0.011	miR-182	3.374	0.046
miR-214	-3.774	0.002	miR-3653	-2.646	0.013	miR-135b	5.456	0.047
miR-139–5p	-3.745	0.003	miR-199a-3p	-2.632	0.014	miR-96	6.117	0.048
let-7c	-3.636	0.003	miR-4684–3p	-2.591	0.015	miR-141	6.211	0.049

**Table 3:** miRNAs that are dysregulated in cancer tissues compared with normal controls by non-paired t-test that have rarely been mentioned in the literature.

miRNA	fold change	P_Value	miRNA	fold change	P_Value	miRNA	fold change	P_Value
miR-4770	-7.407	0.000	miR-24–1*	-4.132	0.002	miR-1979	-2.519	0.019
miR-4510	-5.808	0.001	miR-365	-3.484	0.003	miR-409–5p	-2.222	0.023
miR-99b	-5.566	0.001	miR-4423–3p	-3.125	0.005	miR-26a-2*	-2.222	0.025
miR-4469	-5.222	0.002	miR-628–3p	-3.003	0.006	miR-487b	-2.212	0.026
miR-144*	-4.747	0.000	miR-30c-2*	-2.959	0.007	miR-4443	-2.123	0.028
miR-3195	-4.505	0.001	miR-374b	-2.710	0.010	miR-181a	-1.972	0.032
miR-768–3p	-4.219	0.001	miR-199b-3p	-2.646	0.011	miR-1290	3.341	0.044
miR-125a-5p	-4.184	0.001	miR-4684–3p	-2.591	0.015			

### Comparison of the pattern of expression of miRNAs between Well- and Moderately differentiated adenocarcinoma

Because 7 well-differentiated and 21 moderately differentiated adenocarcinoma samples were included in this study, we proceeded to compare the expression level of the miRNAs in well-differentiated samples and moderately differentiated samples. A total of 58 dysregulated miRNAs were found in moderately differentiated tissues compared with well-differentiated tissues. Fifty of these miRNAs were downregulated significantly in moderately differentiated CRC tissues,while the other 8 miRNAs were upregulated. The fold changes of all 58 dysregulated miRNAs were greater than 2-fold. miR-214 and miR-145 were downregulated more than 10-fold while miR-4797–5p was upregulated 11.9-fold (Table 4).

**Table 4:** miRNAs that could be used to distinguish well- differentiated and moderately differentiated cancers.

miRNA	fold change	P_Value	miRNA	fold change	P_Value	miRNA	fold change	P_Value
miR-214	-10.309	0.001	miR-195	-4.975	0.013	miR-15b	-3.759	0.040
miR-145	-10.000	0.010	miR-152	-4.854	0.040	let-7i	-3.584	0.019
miR-451	-8.929	0.002	let-7c	-4.444	0.024	miR-768–3p	-3.413	0.039
miR-125b	-8.333	0.005	miR-422a	-4.405	0.016	miR-181c	-3.356	0.045
miR-486–3p	-7.463	0.021	miR-515–5p	-4.367	0.021	miR-708	-3.289	0.041
miR-574–3p	-7.407	0.000	miR-28–5p	-4.348	0.007	miR-26a	-3.226	0.050
miR-99a	-7.092	0.013	miR-324–3p	-4.329	0.038	miR-126	-3.185	0.022
miR-1323	-6.993	0.016	miR-505	-4.292	0.032	miR-29a	-3.115	0.019
miR-140–3p	-6.579	0.000	miR-382	-4.219	0.041	miR-10b	-3.096	0.033
miR-143	-6.536	0.036	let-7g	-4.184	0.038	miR-151–5p	-2.857	0.020
miR-409–5p	-6.452	0.001	miR-10a	-4.149	0.014	miR-150	-2.283	0.031
let-7b	-6.250	0.002	miR-16	-4.065	0.018	miR-135b*	6.043	0.037
miR-99b	-6.061	0.011	miR-146a	-4.049	0.009	miR-4795–5p	6.197	0.032
miR-423–5p	-5.952	0.003	miR-27b	-4.016	0.022	miR-4771	8.091	0.041
miR-100	-5.882	0.032	miR-3651	-4.016	0.026	miR-4782–5p	8.442	0.037
miR-342–3p	-5.848	0.002	miR-378i	-4.000	0.029	miR-20a*	8.540	0.038
miR-199b-3p	-5.780	0.004	miR-424	-3.922	0.031	miR-1245b-5p	9.765	0.050
miR-199a-3p	-5.714	0.005	has-miR-16	-3.846	0.023	miR-3133	9.993	0.047
miR-23b	-5.435	0.025	miR-365	-3.802	0.015	miR-4797–5p	11.906	0.017
miR-125a-5p	-5.076	0.016						

### miRNAs that can be used as biomarkers for the diagnosis of colorectal adenocarcinoma

**Fig 1:**
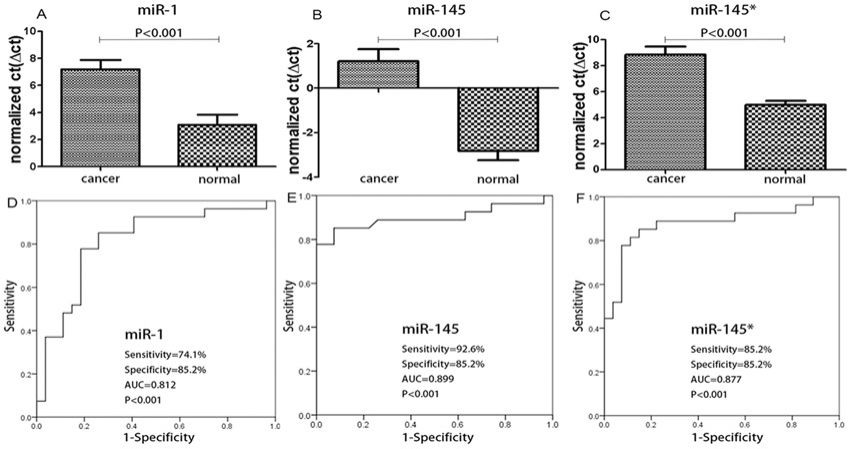
Evaluation of the potential of miR-1, miR-145, miR145* as cancer biomarkers. The results show that miR-1 miR-145 and miR-145* are likely good diagnostic markers with high sensitivity and specificity.

As we presented, miR-1, miR-145 and miR-145* were downregulated more than 10-fold (Table 2, Fig. 1). We then analyzed the specificity and sensitivity of these miRNAs with respect to their ability to distinguish patients with CRC from normal controls by ROC analysis. The results showed that all three miRNAs are likely good biomarkers of Colorectal adenocarcinoma. For miR-1, the sensitivity, specificity and AUC are 74.1%, 85.2%, and 0.812, respectively (P<0.001). With regard to miR-145, the sensitivity, specificity and AUC are 92.6%, 85.2%, and 0.899, respectively (P<0.001). With respect to miR-145*, the sensitivity, specificity and AUC are 85.2%, 85.2%, and 0.877, respectively (P<0.001) (Fig. 1).

**Fig 2:**
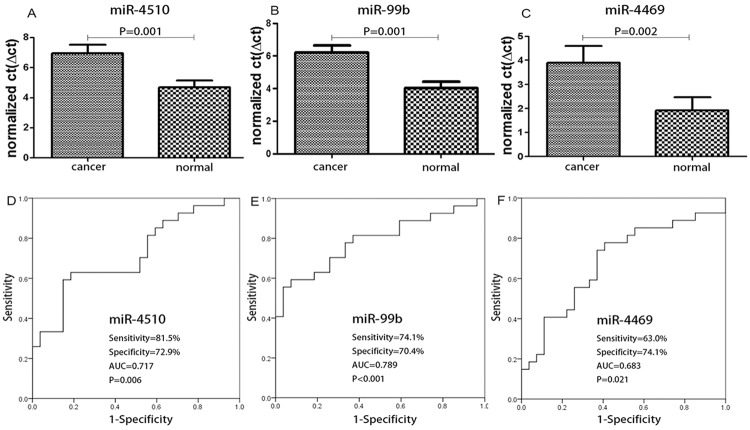
Evaluation of the potential of miR-4510, miR-99b and miR-4469 as cancer biomarkers. The results show that miR-4510, miR-99b and miR-4469 are highly likely to be diagnostic markers with high sensitivity and specificity.

Furthermore, we also analyzed the potential of miR-4510, miR-99b and miR-4469 as biomarkers by ROC analysis. The results showed that the sensitivity, specificity and AUC for miR-4510 are 81.5%, 72.9%, and 0.717, respectively (P = 0.006). With regard to miR-99b, those same values are 74.1%, 70.4%, and 0.789, respectively (P<0.001). With respect to miR-4469, the values are 63.0%, 74.1%, and 0.683, respectively (P = 0.021) (Fig. 2).

### miRNAs that are Dysregulated in cancer correlated with cancer differentiation

**Fig 3:**
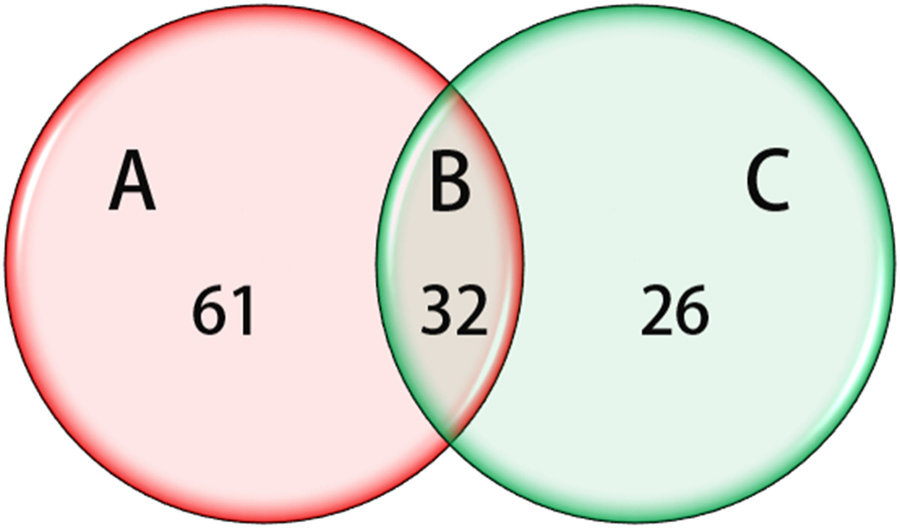
miRNAs that are Dysregulated in cancer correlated with cancer differentiation. (A) Compared with normal tissues, a total of 93 miRNAs that were significantly dysregulated were identified, (B) 58 dysregulated miRNAs were found in moderately differentiated tissues compared with well-differentiated tissues, (C) 32 miRNAs were not only able to identify cancers samples and normal controls, but they were also able to distinguish between well- and moderately differentiated tumors.

**Fig 4:**
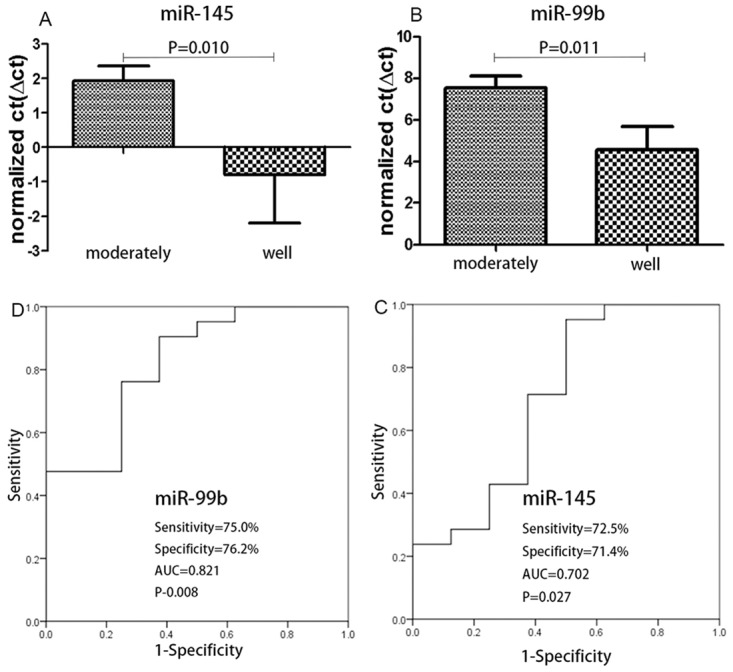
Analysis of the correlation of dysregulated miRNAs (miR-145 and miR-99b) according to the degree of tumor differentiation. The results of the ROC analysis show that miR-145 and miR-99b could not only distinguish cancer samples from normal control samples but also identify well- and moderately differentiated tumors with high sensitivity and specificity.

As mentioned above, we have identified miRNAs that are differentially dysregulated in normal tissue and in adenocarcinoma tissue, and in well- and moderately differentiated cancers. Then, we further investigated whether any miRNAs could be used not only to distinguish cancer tissue from normal tissue but also to distinguish well- and moderately differentiated cancers. Therefore, we have made a connection between these two results. Interestingly, 32 miRNAs were not only able to identify cancers samples and normal controls, but they were also able to distinguish between well- and moderately differentiated tumors (Table 5, Fig. 3). MiR-145 and miR-99b, as mentioned above, performed well in the biomarker assay for the diagnosis and classification of cancer (Fig. 4).

**Table 5:** miRNAs that were not only able to distinguish cancer patients from normal controls but also able to distinguish well-differentiated and moderately differentiated cancers.

miRNA	fold change	P_Value	miRNA	fold change	P_Value	miRNA	fold change	P_Value
miR-145	-16.393	0.000	let-7c	-3.636	0.003	miR-422a	-2.793	0.010
miR-143	-7.194	0.000	let-7b	-3.623	0.003	miR-199b-3p	-2.646	0.011
miR-99b	-5.566	0.001	miR-365	-3.484	0.003	miR-199a-3p	-2.632	0.014
miR-99a	-5.348	0.001	miR-28–5p	-3.401	0.003	miR-10b	-2.577	0.016
miR-451	-5.051	0.001	miR-574–3p	-3.300	0.004	miR-140–3p	-2.558	0.016
miR-23b	-4.630	0.001	miR-378i	-3.185	0.004	miR-10a	-2.342	0.021
miR-100	-4.608	0.001	miR-27b	-3.058	0.006	miR-409–5p	-2.222	0.023
miR-125b	-4.386	0.001	miR-26a	-2.985	0.007	miR-181c	-2.058	0.028
miR-768–3p	-4.219	0.001	miR-126	-2.950	0.009	miR-150	-1.976	0.030
miR-125a-5p	-4.184	0.001	miR-195	-2.950	0.009	let-7i	-1.876	0.032
miR-214	-3.774	0.002	let-7g	-2.817	0.009			

**Fig 5:**
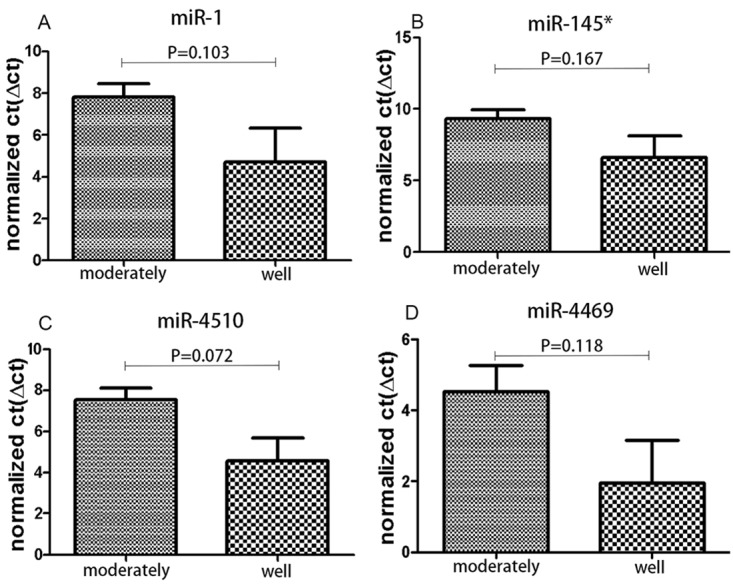
Analysis of the expression levels of miR-1, miR-145*, miR-4510 and miR-4469 between well- and moderately differentiated tumors according to a non-paired t-test. The results show no obvious differences in the expression levels of miR-1, miR-145*, miR-4510 and miR-4469 between well- and moderately differentiated tumors (*P*>0.05).

However, other miRNAs such as miR-1, miR-145*, miR-4510, and miR-4469 were dysregulated, but the differences were not statistically significant (Fig. 5).

### Independent sample test to confirm the previous microRNA profile results

To confirm the reproducibility of our findings, we performed an independent sample test for the expression levels of miR-1, miR-145, miR-145*, miR-99b, miR-4510 and miR-4469. The results of this test demonstrated that the expression levels of the above-mentioned 6 miRNAs are downregulated in CRC, which is consistent with our previous findings (Table 6).

**Table 6:** Expression levels of miR-1, miR-145, miR-145*, miR-99b, miR-4510, and miR-4469 in an independent sample test.

miRNA	fold change	P_Value	miRNA	fold change	P_Value
miR-1	-12.821	0.009	miR-99b	-5.556	0.004
miR-145	-11.494	0.000	miR-4510	-6.667	0.008
miR-145*	-15.385	0.001	miR-4469	-4.367	0.030

## Discussion

Colorectal adenocarcinoma represents a lethal disease that has attracted substantial attention, especially because the current screening methods for Colorectal adenocarcinoma are still limited by unsatisfactory sensitivity and specificity. MicroRNAs have been shown to directly impact tumor growth, tumor cell proliferation, and tumor invasion [10], and they have also been demonstrated to be potential biomarkers of Colorectal cancer. Many dysregulated miRNAs were identified in colorectal cancer specimens. However, no research has been conducted on a comparison of the miRNA profiles among different classifications of tumors. Additionally, previous studies have reported that miRNA profiles can contribute to the diagnostic and prognostic classification of human malignancies [14–16]. CRC contains a variety of histologic tumor types, for example: (1) adenocarcinoma; (2) gland scale cancer; (3) small cell carcinoma; (4) carcinoid; (5) anaplastic carcinoma; and (6) squamous cell carcinoma, among others. According to reports from the 1980s to the 1990s, the proportion of Colorectal adenocarcinoma has increased from 82.1% to 85.6% in China [17]. Therefore, Colorectal adenocarcinoma was the cancer type selected for our study. In the present study, we first compared the expression level of 1547 miRNAs in Colorectal adenocarcinoma tissues with that of paired normal tissues and identified 93 miRNAs that were significantly dysregulated in Colorectal adenocarcinoma relative to normal tissues (*P*<0.05). Of these, miR-1, miR-145 and miR-145* were downregulated more than ten-fold, and this result was consistent with the results of previous studies. Bao Y and his colleagues reported that miR-145 was significantly downregulated in colorectal cancer tissues [18] and that the targets of miR-145 are involved in cell cycle and neuregulin pathways [19]. In addition, they showed that the upregulation of miR-145 could improve the ability of colorectal cancer cells to migrate and invade; the mechanism of the promotion of migration and invasion is associated with the stabilization of Hsp-27, a protein that plays an important role in the promotion of metastasis [20]. Furukawa S and his colleagues demonstrates that miR-1 is downregulated in colorectal tumors and that miR-1 has the potential to suppress NOTCH3 expression through direct binding to its 3'-UTR region. It is also known that NOTCH signaling plays critical roles in colorectal tumorigenesis [21]. Downregulated miR-1 can cause tumor suppressor effects in colorectal cancer by the direct downregulation of the MET oncogene both at the RNA and the protein level. Re-expression of miR-1 leads to a MET-driven reduction of cell proliferation and motility, which suggests that the **downregulation of** miR-1 is one of the events that might enhance colorectal cancer progression [22]. For the most part, this result was verified by an independent sample test (Table 6). Moreover, we analyzed the potential of miRNAs as cancer biomarkers by ROC analysis, and the result revealed that all three miRNAs are suitable as diagnostic biomarkers, as their sensitivity, specificity, and the value of AUC are all greater than 0.7 [23]. In addition, 23 of these miRNAs have been scarcely reported until now (Table 3) with the exception of miR-4770 and miR-3195, which have been mentioned in our previous research [12]. Of these miRNAs, miR-4510, miR-99b and miR-4469 were downregulated more than 5-fold. Thus, we analyzed the sensitivity and specificity of these miRNAs by ROC analysis, and the results showed that they are capable of distinguishing cancer patients from normal controls. Next, we wanted to analyze the difference between well- and moderately differentiated cancers, and we found that expectedly, 58 miRNAs showed differential dysregulation (Table 4). Furthermore, to find miRNAs that could not only distinguish cancer patients from normal controls but that could also distinguish well-differentiated cancers from moderately differentiated ones, we formed a connection between the two results mentioned above. Surprisingly, 32 miRNAs met this criteria (Table 5). As shown, miR-145 and miR-99b were a part of this relationship. Their expression levels changed more than 5-fold, and the ROC analysis showed that miR-145 and miR-99b were able to distinguish well-differentiated cancers from moderately differentiated ones. However, the other 4 miRNAs (miR-1, miR-145, miR-4510 and miR-4469) were also dysregulated, but the differences were not statistically significant (Fig. 5).

On the one hand, the expression profiles of the miRNAs in our study confirm some existing findings, but on the other hand, we also obtained some contrasting results. For example, the expression patterns of miR-96 and miR-135b that are reported in Hamfjord’s study are the same as in our study, but the expression of profiles of miR-552 and miR-549 are different between the two studies. These two miRNAs were significantly upregulated in Hamfjord’s study while they were dysregulated in our study; the differences were not statistically significant [11]. Li J and his colleagues found that the expression of miR-429 was upregulated in human colorectal cancer (CRC) tissues, and the increased expression was significantly associated with tumor size, lymph node metastasis and poor prognosis. This is consistent with our findings [24]. However, in contrast to our studies, Sun Y reported that miR-429 was significantly downregulated in colorectal carcinoma (CRC) tissues and cell lines [25]. This phenomenon may be due to: 1) differences in the ethnicities of the patients, locations, genes and/or screening criteria, and these discrepancies might be settled by the establishment of unanimous screening standards in the near future; 2) a variety of techniques that are used to examine the expression of selected miRNAs, so cost-effective, simple and proven techniques should be found; 3) the defects of experimental design that need to be slowly improved. Due to these potential explanations, more studies are needed in order to reduce future errors.

Our study has several weak points that should be acknowledged. The most significant shortcoming of our study is the limited number of samples that were included in the experiment. Therefore, the results of the present study require validation by further investigation of larger CRC patient cohorts. However, despite its limitations, this is the first analysis of miRNA profiles based on degrees of differentiation, and we obtained some meaningful results. These results are vital for future research because it is important to know whether miRNA profiles are affected by the degree of tumor differentiation before miRNAs can be used as biomarkers in clinical diagnosis.
